# Using a Virtual Community (the Health Equity Learning Collaboratory) to Support Early-Stage Investigators Pursuing Grant Funding

**DOI:** 10.3390/ijerph15112408

**Published:** 2018-10-30

**Authors:** Meldra Hall, Jeffrey Engler, Japera Hemming, Ernest Alema-Mensah, Adriana Baez, Kimberly Lawson, Alexander Quarshie, Jonathan Stiles, Priscilla Pemu, Winston Thompson, Douglas Paulsen, Ann Smith, Elizabeth Ofili

**Affiliations:** 1 Clinical Research Center, Morehouse School of Medicine, Atlanta, GA 30310, USA; jhemming@msm.edu (J.H.); klawson@msm.edu (K.L.); 2 Council of Graduate Schools, Washington, DC 20036, USA; jengler@cgs.nche.edu; 3 Department of Community Health and Preventive Medicine, Morehouse School of Medicine, Atlanta, GA 30310, USA; eamensah@msm.edu (E.A.M.); aquarshie@msm.edu (A.Q.); 4 University of Puerto Rico, San Juan, PR 00936-5067, USA; adriana.baez@upr.edu; 5 Department of Microbiology, Biochemistry, and Immunology, Morehouse School of Medicine, Atlanta, GA 30310, USA; jstiles@msm.edu; 6 Department of Medicine, Morehouse School of Medicine, Atlanta, GA 30310, USA; pipemu@msm.edu; 7 Department of Physiology, Morehouse School of Medicine, Atlanta, GA 30310, USA; wthompson@msm.edu (W.T.); eofili@msm.edu (E.O.); 8 Department of Pathology & Anatomy, Morehouse School of Medicine, Atlanta, GA 30310, USA; dpaulsen@msm.edu; 9 University of Alabama, Birmingham, AL 35294, USA; annsmith@uabmc.edu

**Keywords:** grant writing, underrepresented groups, collaboratory, submission habits

## Abstract

Junior investigators often have limited access to networks of scientific experts and resources that facilitate competitive grant submissions. Since environments in which scientists are trained are critically important for long-term success, we built and tested a virtual environment for early-stage investigators (ESIs) working on grant proposals. The aim of this study was to evaluate the virtual community’s influence on grant submission patterns among participants from underrepresented groups. As part of a grant writing coaching model, junior investigators were recruited into a professional development program designed to develop competitive grantsmanship skills. Designed by the Research Resources and Outreach Core (RROC) of the National Research Mentoring Network (NRMN), the Health Equity Learning Collaboratory (EQ-Collaboratory) provided a virtual community for social support, accountability, constructive feedback, and access to peer networks to help investigators overcome barriers to grant submission. This study assessed differences in outcomes for participants who completed the training within the EQ-Collaboratory compared to those who did not. The analyzed data revealed a statistically significant difference in the average time to submission for participants enrolled in the EQ-Collaboratory. EQ-Collaboratory ESIs submitted proposals 148.6 days earlier, (*p* < 0.0001). The results suggest that a supportive virtual environment can help investigators more quickly overcome barriers to grant submission.

## 1. Introduction

Since investigators who come from diverse backgrounds bring a set of distinct experiences to investigating research problems, each one has something unique to contribute to both the conception of the research strategy and the writing process for presenting the findings. Therefore, such diversity of thought and experience is a substantial benefit, particularly when scholars are engaged in scholastic discussions [1].

Early-stage investigators (ESIs) from groups underrepresented in the STEM workforce generally have limited access to an environment that supports their motivations to progress in biomedical research. These environments most often include both networks of collaborators and scientific experts in their discipline and access to resources that promote competitive grant submissions [2]. These challenges are even greater among investigators from underrepresented groups (URGs) in the biomedical workforce. These investigators are often less represented in leadership and full professorship, and, as noted, have been less likely to be awarded R01s [3]. The social context in which these investigators are trained is critical for their professional development, promotion, and retention [4]. For example, socially affirmative environments result in stronger collaborations, better solutions, and increased productivity [5]. In the academy, research productivity, as measured by peer-reviewed publications in high-impact journals and successful receipt of grant awards, is often one of the criteria for promotion and tenure [6]. As such, improving competitive grant submission is a critical part of the strategy to improve diversity in STEM, and diversity is linked to better scientific inquiry and discovery.

Investigators from URGs cite various real and perceived barriers that impede the development and submission of grant applications, including but not limited to inadequate training and development, mentoring, and the lack of institutional support [6]. Given that diversity in the biomedical research workforce transcends geographical and spatial boundaries, we built and tested a virtual support environment for early-stage investigators, predominantly from underrepresented groups, working on grant proposal development, one of the key markers of career progression. Evidence from biomedical junior faculty and pos-doctoral scholars from URGs suggests that social support and career coaching are essential for career progression [7]. Therefore, the frame for this paper is an environmental professional development solution to address challenges in grant submission for investigators from underrepresented groups. Because little research has been done at the faculty level on virtual social environments to support professional development, this is a new investigation and research area.

To address the shortage in federal funding of minority ESIs and the need for professional development through mentoring, the National Research Mentoring Network (NRMN) was launched by the National Institutes of Health (NIH). The NRMN has played a key role in professional development and mentoring of participants through grant writing programs. The Research Resources and Outreach Core (RROC) is part of the NRMN initiative and works to leverage the research infrastructure of the Research Centers at Minority Institutions (RCMI) Program and partnering Clinical and Translational Science Award (CTSA) institutions to deliver initiatives that attract diverse scholars across career stages for participation in NRMN programming and to enhance mentoring capacities across target institutions. The RROC launched Strategic Empowerment Tailored for Health Equity Investigators (SETH) as its primary program to address the diversity shortage. SETH is a grant writing coaching group program, adapted from the Northwestern University Grant Coaching Group Model that targets junior faculty, post-doctoral scholars, and prospective NIH investigators interested in participating as a mentee, to learn rhetorical patterns and effective writing techniques for NIH-style proposals [8].

The overarching purpose of this work is to understand how expanding external networks and providing online support can strengthen the research and professional environments experienced by biomedical faculty from underrepresented backgrounds, to increase the diversity of the STEM workforce. We explored possible differences in outcomes (as measured by grant submission and time spent on grant writing) for participants who did or did not have access to the curated virtual online community of other URG researchers and online resources, available in the collaboratory.

We hypothesized that because the Health Equity Learning Collaboratory (EQ-Collaboratory) provides social support and career coaching, participants in the collaboratory are more likely to submit their grant proposal over a shorter time frame. Preparing and submitting grant applications is a crucial step in career progression for biomedical faculty. Our second hypothesis tested whether EQ-Collaboratory intervention increased the rate of proposal submissions.

### 1.1. Theoretical Framework

To formalize the activities conducted in the EQ-Collaboratory, the Communities of Practice framework [9] was employed to guide the structuring of this social environment. The main constructs in this theory are Domain, Community, and Practice, each of which is present in our program design.

- Domain (the online collaboratory) is the technology that facilitates dialogue which promotes learning and information transfer.
- Community (ESIs, coaches, coaching directors, and supporting research staff) refers to those individuals who engage and collaborate for learning to occur.
- Practice (ongoing and continued practice and modeling of the grant writing process) represents the knowledge that is acquired as a consequence of the engagement.

Although we used the term “collaboratory” in the context of biomedical research, this term is generally used to describe an online community of people who work remotely on shared or collaborative tasks. The literature notes that the tasks are completed just as efficiently as if they were present in the same room [10]. One study looked at virtual communities within the context of marketing [11]. In their highly cited article, *Distance Matters*, Olsen and Olsen discuss four components for successful virtual engagement while working in disparate locations. The four components include: common ground, coupling in work, collaboration readiness, and collaboration technology readiness [10]. Although common ground—the shared knowledge collaborators have in common and the first of four components for virtual interaction—is a critical concept, it is limited in definition and cannot be extrapolated to the structure of the collaboratory. The second concept, coupled in work, is inadequate since it requires that task completion be dependent on the entities of the group. Although each ESI benefits from the contributions made by all group members, the presence of all group members is not a requisite for grantsmanship progress to occur. Lastly, technology readiness assesses the level of expertise which individuals have when adopting the new technology. Variance within uptake was insignificant since ESIs were given training prior to engaging with the online platform. Despite functioning as critical components, these three concepts were insufficient for the structure of the social environment we designed. Consequently, we chose collaboration readiness—the motivation for coworkers to collaborate—as the basis for this intervention because of its premise of information sharing within a collaborative context.

The participants recruited to SETH collaborated with peers at similar career stages and had discussions to improve the competitiveness of their grant applications while in the grantsmanship coaching groups. As a result, the EQ-Collaboratory was created as a virtual community in which scholars who are interested in health equity research as well as basic science research could engage in scientific discussions with peers and with a mentor within their coaching groups.

### 1.2. How the Experiment is Unique and/or Builds on Existing Literature

Since the EQ-Collaboratory was designed to provide social support, this study seeks to understand how the social support in a virtual environment influences the proposal submission habits of participants from underrepresented groups. This manuscript will present preliminary evidence to document the outcomes of ESIs, after participating in this online environment to support their grant writing skill development and networking. Since some ESIs may lack access to the professional development resources within their home institutions, the EQ-Collaboratory creates an environment that supports the professional development of these participants.

## 2. Materials and Method

Participants were recruited into a grant writing program designed to expose them to a specific writing style. The RROC used several culturally competent outreach strategies to enhance recruitment efforts, including but not limited to: (1) building relationships with the target audience; (2) understanding the needs of the target audience; (3) identifying important issues unique to the target audience; (4) maintaining a presence in the targeted community; and (5) partnering with diverse organizations for recruitment. Although recruitment strategies evolved over the two years of this project, these underlying themes remained constant. With each new cohort, the implementation of activities evolved.

### 2.1. Organization of the Intervention and Recruitment of ESIs for the First Two Groups (Pre-Collaboratory)

The first two cohorts, coaches, along with ESIs, were recruited through the RROC External Advisory Board members, many of whom were principal investigators of the Research Centers at Minority Institutions (RCMIs), the Clinical and Translational Science Awards (CTSA), and other partnering organizations. The recruitment efforts initially leveraged resources through RCMIs in which principal investigators were asked to nominate potential participants that would benefit from a tailored grantsmanship model. Since ESIs came from both the mainland and the island of Puerto Rico, ESIs were also recruited by one of our Associate Directors (AB), a faculty member at the University of Puerto Rico who had substantial knowledge of junior faculty located in research institutions on the island. These recruitment efforts led to a cohort of ESI participants mostly from RCMIs and the Puerto Rico University System, with a few others from non-RCMI institutions.

In addition to recruiting ESIs, the research team at SETH recruited coaches, senior faculty with previous experience in developing and securing NIH funding, to assist with training ESIs.

After these ESIs had been recruited, they were encouraged to apply to the training program. During the application process, the review committee, made up of RROC team members (MH, JE, JH, KL), used an assessment tool to evaluate participants. Trainees were evaluated based on their self-reported research training and experience, writing preparedness, and fit for the program. Each applicant was assessed by the review committee using a 4-point Likert scale that ranged from *strongly agree* to *strongly disagree* to determine the extent to which the applicant was ‘ready to write’. Although the applicant was accepted based on the score they received, our sample of participants was not selected via random sampling of applicants. The pre-interviews were designed to identify those most likely to write an application within the time frame of the intervention. We undertook this selection to make the best use of available resources needed to support applicants most likely to be successful. The selection criteria were subjective because we were identifying “readiness to write”.

Upon being accepted into the program, ESIs were then matched with a coach, trained in the art of developing grantsmanship skills in ESI, according to their discipline and research area, to form a coaching group with 3–4 other ESIs. The coaching group met inperson for 2–3 days during which a specific grant writing workshop that focused on the rhetorical patterns of writing was delivered, and then met virtually for 4–6 months, giving and receiving feedback on the proposal, particularly the specific aims page. These coaching groups function as guides to support ESI professional development through scientific discussions.

The grantsmanship coaching group training, designed to provide trainees with the opportunity to engage in dialogue and learn the art of crafting NIH-style proposals, was initially launched with participants in groups 1 and 2 (Pre-Collaboratory [Pre-CC]) in May and November 2016 at Morehouse School of Medicine in Atlanta, Georgia and in San Juan, Puerto Rico at the University of Puerto Rico (UPR) Medical Science Campus, respectively. Since participants in the Pre-CC were trained solely using the coaching group model, they had no access to the virtual community and supplemental resources throughout their coaching season.

After conducting the first two cohorts, evaluations revealed some of the barriers to committing to the program and completing the grant writing. For example, ESIs experienced inconsistent engagement from coaches, ESIs had to find mentoring teams after being accepted into the program and this led to a change in the direction of their project, some ESIs were not well-positioned to submit a grant, and some had not collected sufficient data to support a grant submission. Additionally, the barriers included an environment that facilitated accountable coaching and access to readily available online NIH resources.

### 2.2. Development of the Post-Collaboratory Intervention and Virtual Community

**Figure A1:**
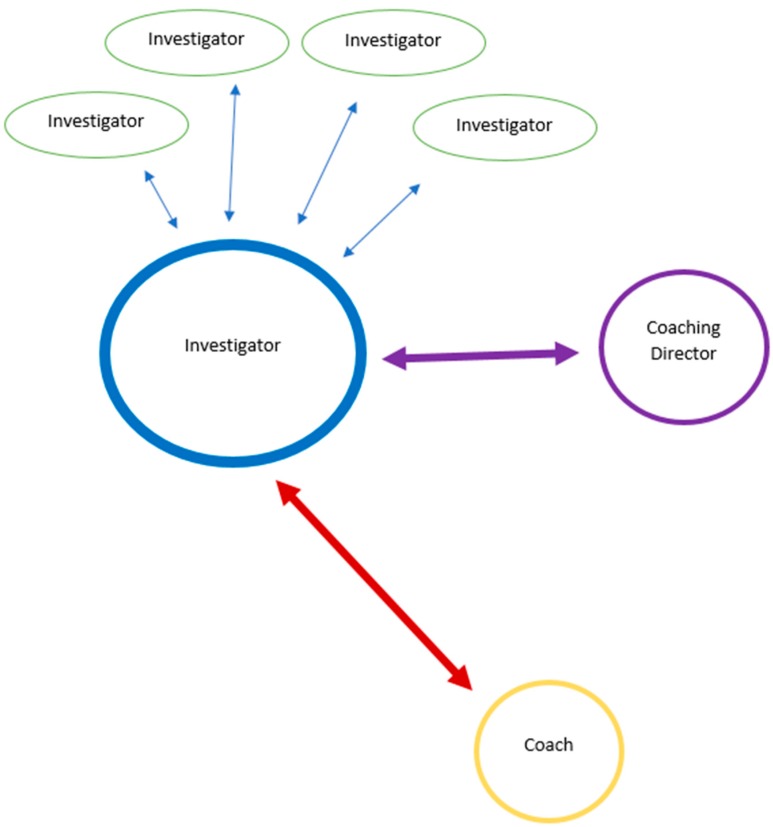
Diagram of social network in coaching group in collaboratory.

As a response to these ongoing barriers, the EQ-Collaboratory was introduced in the subsequent cohorts to support ESI in their structured networks (shown in Figure A1) and increased exposure to resources that would provide aid in the grant writing process. Additionally, the EQ-Collaboratory created a space for a more structured environment that facilitated consistent coaching and accountability for both junior investigators and coaches. Investigators in collaboratory-facilitated coaching groups had scheduled and tracked bi-weekly coaching sessions. NIH resources, made readily accessible, allowed junior investigators to glean grant-related information in condensed time. Specifically, these resources included: (1) online office hours held by the coaching director (JE) to have virtual sessions with the participants to answer questions about their grant; (2) a grant writing webinar to provide budgetary expertise to participants when preparing to draft a budget; (3) information containing NIH resources (i.e., mp3 recordings on various NIH grant writing topics); (4) links to new RFAs that the NIH released; (5) notifications for NRMN monthly webinars; (6) helpful NRMN career-development videos; and (7) NIH Loan Repayment opportunities. The Collaboratory also benefits trainees by working as a scheduling tool, keeping up with tasks to be completed (creating accountability to complete writing tasks on time), and storing shared files. Furthermore, online access to expert advice from the coaching director during scheduled office hours was now an option along with a webinar to help with the grant submission process. The research team at RROC was interested in back-end analysis of coaching interactions and the collaboratory afforded this function.

These resources were afforded to the post-collaboratory cohorts (Post-CC) which consisted of two groups of ESIs, with in-person meetings held in May 2017 at Morehouse School of Medicine in Atlanta, Georgia and in October 2017 in Washington, D.C. at the Wardman Park Hotel, during the RCMI Translational Science Conference. Given that the in-person meetings were followed by 4–6 months of virtual coaching in the EQ-Collaboratory, the two Post-CC utilized the support offered by the virtual community within the Collaboratory during this timeframe. Furthermore, since participants were in the collaboratory, a dedicated research assistant (MH) could inform trainees of assessments that were about to go out and more closely follow up with the scholars about incomplete assessments.

For the post-collaboratory cohorts, recruitment was facilitated by our RCMI and CTSA collaborators, NRMN cores, and by the partnership with Building Infrastructure Leading to Diversity (BUILD) Institutions. Since the in-person meeting held in Washington, D.C. was in collaboration with the RCMI Translational Science Conference, recruitment efforts took advantage of the meeting attendees and referrals. The Principal Investigator (EO) also used her network to recruit scholars. The coaches were recruited through the efforts of the coaching director (JE).

### 2.3. Enhanced Screening of Applicants for Readiness

The EQ-Collaboratory (used interchangeably with “the collaboratory”) was a key feature of the post-collaboratory cohort. In addition to the introduction of the EQ-Collaboratory, other modifications included: improving recruitment strategies; integrating a more stringent review process; expanding the in-person training to three days; requiring a signed mentor-mentee compact agreement between coach and trainee; introducing the SETH coaching director; and implementing a program called “Shark Tank” designed to support scholars re-submitting a previously unfunded application.

The review committee, which consisted of multiple individuals, reviewed applications, and invited applicants for an interview prior to acceptance into the program. The review committee conducted video interviews with each applicant after the coaching director’s initial screening. The collaboratory-facilitated interviews were held to assist in evaluating applicants based on existing mentoring teams and institutional support.

Being that the EQ-Collaboratory supported both the pre-session interviews and the coaching groups, the research questions therefore were “Are ESIs who engage with the online collaboratory more likely to submit grant applications? Do ESIs who engage with the collaboratory have a shorter submission window?”

### 2.4. The Compact Between ESI and Coach

The compact, given to both coach and ESI to document their commitment to each other and to the proposal preparation process, emphasized the privacy and confidentiality expectations of the group process and served to build trust between the coach and their assigned ESI. In addition to leading the face-to-face training at Morehouse School of Medicine, the coaching director monitored the activities of the coaches and their subsequent interactions with their respective ESI in the coaching groups. In this cohort, there were participants who had already submitted proposals and had summary statements from review panels. To help these ESIs revise a previously unfunded proposal, “Shark Tank,” led by one of our Associate Directors (JS), was implemented to provide tailored coaching to ESIs who were re-submitting proposals.

### 2.5. Trainees were Placed in a Virtual Community with Other Participants

We evaluated the differences in SETH Pre-Cc and Post-CC outcomes. Although these previously mentioned variables were introduced in the Post-CC, we were unable to control for them in the statistical analysis.

The measures ‘time to grant’ and ‘submission rates’ were used to assess the EQ-Collaboratory Outcomes. Time to grant was equal to the grant submission date minus the program start date. This variable was measured in days. Submission rates were measured across 6 months.

We used a 6-month post assessment as our timeframe, in order to standardize the submission period for both cohorts. During the course of the coaching groups, there were inconsistencies between intended grants and actual grant mechanisms submitted. The scholars’ mechanisms were slightly altered based on discussions with and expert advice from their primary coach. Many scholars arrived thinking they were ready to submit a particular grant mechanism but after a conversation with their coach, they quickly realized they were not as ready to submit that proposal. Since trainees tended to underestimate their readiness for a particular proposal, coaches often times encouraged ESI to submit grants for which they were more competitive.

This study was approved by the Institutional Review Board (IRB) at Morehouse School of Medicine and the IRB study protocol number is 774520.

### 2.6. Statistical/Data Analysis

Data analysis was run to obtain univariate and multivariate analyses. Univariate analysis was run using SAS 9.4 to obtain descriptive variables of the coaches’ trainees in each coaching group. Variables included interview, collaboratory, and number of submissions. The coaching groups that were led by coaches who participated in both pre-collaboratory cohorts and post-collaboratory cohorts were separated out and included in each cohort analysis in which they participated. Descriptive analysis for race, gender, career stage and type of institution also was run.

Stata was used to run a t-test procedure to compare the two means to determine if there was a difference in the time to submit and submission rate between the Pre-CC and Post-CC. The confidence interval was used to determine the direction of the difference between the cohorts. T-test is reported to determine if there is a difference, and we report confidence intervals (the direction of the difference) to test our hypotheses.

Multivariate analysis was run to control for covariate which the literature had established to determine true associations.

## 3. Results

Of the 113 trainees and 26 distinct coaching groups, 13 coaching groups were in the Pre-CC and 13 were in the Post-CC. Of the SETH-trained ESI, 50% (56) were in the Pre-CC and 50% (57) were in the Post-CC. Within the Pre-CC, the races/ethnicities represented included Asian (16%), Black (38%), Hispanic/Latino (36%), White (9%) and other-Arab (2%). For the Post-CC, the races/ethnicities were more widely distributed among Asian (14%), Black (46%), Hawaiian-Pacific Islander (2%), Latino (9%), Native American (2%), White (26%), and other-Arab (2%). The gender distribution among the trainees was approximately a 3:1 ratio with the Pre-CC having 75% female and 25% male, while the Post-CC had 63% female, 36% male, and 2% other. Most of the trainees were in the post-doc (30%-Pre-CC; 20%-Post-CC) and Assistant Professor (54%-Pre-CC; 61%-Post-CC) career stage, with a few others falling in the Associate Professor (12%-Pre-CC; 13% Post-CC), Professor (0% Pre-CC; 2% Post-CC), and Other/Researcher (2% Pre-CC; 2% Post-CC) stage. With regards to ESI institution, 50% were from RCMIs and 76% (16) of the 21 RCMIs were represented in the SETH cohorts.

Overall, 59 trainees submitted a proposal: 31 (55%) submissions in the Pre-CC and 28 (49%) in the Post-CC. However, the Pre-CC included 18 months of data collection while the post-collaboratory cohort consisted of 6 months of data collection. When the data were standardized, 42 ESIs submitted within the first sixth months of their cohort starting; the Pre-CC had 14 (25%) submissions within the first 6 months while the Post-CC had 28 (49%) submissions in the same timeframe (*p* < 0.002). The submission rate for the Pre-CC was 24.4 ± 19.06 (95% confidence interval [CI] 13.42–35.39); the Post-CC had a 47.0 ± 22.44 submission rate (95% CI = 38.56–56.31). For time to submit, the Pre-CC took 249.4 ± 55.08 on average to submit their grant proposals, with a range of 7–647 (95%CI = 234.64–264.15), while the Post-CC took on average 100.8 ± 50.06 (*p* < 0.0001) days to submit, with a range of 17–189 (95%CI = 87.26–114.33). The Pre-CC took a median of 251 days to submit and the Post-CC took a median of 100 days. ESIs with access to the online virtual community (the Post-CC) were more likely to submit and therefore demonstrated better outcomes as measured by both grant submission rates and time taken to submit the research proposal even after adjusting for race, gender, career stage, institution, and coaching group.

Table 1 shows the outcomes from regression model after controlling for variables known in the literature to contribute to submission rates: gender, race, and career stage. The unadjusted odds ratio shows that investigators in the post-collaboratory cohort were almost 3 times more likely to submit (*p* < 0.009). Additionally, to evaluate the nesting effect of the coaching groups in our data, we analyzed for an association between coaching group and likelihood of submission but there was no significant association. The adjusted odds ratio revealed the post-collaboratory investigators were almost 5 times more likely to submit within the first 6 months.

Table 1 demonstrates that the odds of investigators submitting in the post-collaboratory cohort are higher than the odds for the pre-collaboratory cohort investigators.

Since the literature shows that the URG are less likely to re-submit, we designed a program to address this perceived barrier (Ginther, 2011). As an additional support for participants re-submitting a previously unfunded proposal, “Shark Tank” was introduced for the post-collaboratory participants. ESIs who submitted a summary statement were placed in the Shark Tank coaching group, which was led by the RROC Associate Director (JS), an experienced researcher and grant writer. Five participants made up the Shark Tank group. To date, two of the five participants have submitted their grant proposals and one of these proposals has been funded.

## 4. Discussion

ESIs investigating health equity issues bring a diverse set of experiences that adds to their engagement with the collaboratory. These scholars have unique characteristics and experiences they bring to research and to the grantsmanship writing group cohorts but unfortunately they may not be exposed to the network needed to write competitive proposals. After conducting the first two pre-collaboratory cohorts, analysis of the evaluation data showed that the pre-collaboratory cohort continued to experience barriers to grant submissions despite their participation in the grantsmanship coaching group. This cohort was conducted without the availability of the online support community (the EQ-Collaboratory), and we observed that grant submission rates were relatively low. Consequently, the length of time participants took to submit their proposal was longer than the projected six months predicted by the grantsmanship coaching model. We observed that the coaching model was insufficient in helping URGs in our cohort submit research grant proposals in a timely manner.

The key findings in this study were that 28 of the post-collaboratory trainees submitted within the first six months of the intervention, showing a mean submission rate of 47%, while only 14 of the pre-collaboratory ESI submitted in the same timeframe, with a 24.4% mean submission rate. Another important finding was the submission rate of all the submissions made. For the Post-CC ESI, almost 90% of the submissions were within six months of the start of the coaching group, while only half that percentage (45%) of all the submissions were submitted within the same timeframe in the pre-collaboratory cohort. These data suggest that the ESIs in the collaboratory were more likely to submit a proposal than those who did not have access to those resources.

Additionally, the times to submission outcomes are worth noting. The mean time it took ESI to submit in the Pre-CC (249.4 days) was almost three times the time it took the Post-CC to submit (100.8 days). Since fewer of the Post-CC (39%) had unsuccessful prior grant submissions compared to their Pre-CC peers (52%), the previous grant submission would not contribute to a shortened overall time to submission for the Post-CC. This outcome shows that in addition to being better positioned to submit their proposals, ESIs in the collaboratory were more likely to take fewer days to complete and submit their proposals. Most significantly, the Post-CC was approximately five times more likely to submit after adjusting for covariates.

Despite the optimistic findings, there was an unexpected outcome of the interview process: building trust and engagement with each applicant. During the interviews with the applicants, these personal interactions led to honest feedback on their readiness and where they were in the process. They also were clearer on where they were as far as their career stage was concerned, noting whether they were an instructor, research assistant professor, post-doc, or assistant professor. The applicants seemed much more open and honest about their goals, contributions, and expectations of the program when they were engaging in face-to-face dialogue during the video conference that took place in the EQ-Collaboratory. This allowed the review team to have a much deeper understanding of each applicant’s motivations and expectations.

In addition to the previously mentioned outcome, another unanticipated finding was observed: participants in the post-collaboratory cohort was also more responsive than their pre-collaboratory peers in completing the various evaluations, both at the end of the intervention and with the pre-session, midpoint, and post-session evaluations. For the post-collaboratory cohort, all coaching group engagement along with the frequency of the meetings was tracked in the collaboratory. Coaches met on a bi-weekly basis through video chat conferencing provided in the EQ-Collaboratory. In addition to the meetings, trainees engaged in office hours with the coaching director, attended a pre-grant submission webinar, and accessed NIH resources that were available through the collaboratory.

Through this study, we explored the impact that the Health Equity Learning Collaboratory had on early-stage investigators. Since 69% of these participants were underrepresented minorities, it was critical to understand the outcomes of an expanded network and additional access to resources not ordinarily found in a scholar’s network. These results suggest that the network of the coaching group and the resources provided in the online collaboratory (i.e., the video conferencing, webinars, links to NIH information) could improve both the rate of the grant submission and the time taken to submit the grant.

The literature supports the outcomes of this study in that an intervention provided to a group of underrepresented minorities with challenges in accessing institutional resources, can help increase grant submission rates (Viets et al., 2009). However, the study by Viets et al. is limited because it relied solely on in-person meetings to assess outcomes and did not explore how a virtual community could impact submission rates when scholars work remotely in accomplishing tasks essential to their professional development and career progression.

In addition to implementing the collaboratory, there were two other post-collaboratory changes made: (1) the coaching director was appointed to facilitate the collaborations between trainee and coach; (2) a more stringent review process was initiated; and (3) the compact agreement. In the statistical analysis of the raw data, we could not disaggregate the contribution of variables for the changes we made in the post-collaboratory cohort, i.e., engagement with a coaching director, and implementing a more stringent review process of applicants. Therefore, the statistical analysis did not control for all the changes made in the post-collaboratory cohort. However, the analysis does control for factors likely to influence outcome variables, including gender, race, and career stage. The results suggest that there is a strong correlation between those in the Post-CC and higher submission rates along with a significantly diminished time to submission.

With respect to the post-collaboratory recruitment strategy, the outreach was tailored to the ESI targeted. Although the PI used her network in the Post-CC, the RCMI institutional leaders involved in the Pre-CC recruitment strategy were also in the PI’s network. Although the PI’s network has the potential to influence participants’ engagement level, it would not account for the ensuing Post-CC submission outcomes.

This review process assessed the variables influencing the applicant’s readiness to submit a grant. Despite these limitations, this study brings strengths to the body of literature, including having two equivalent pre- and post-collaboratory cohort sizes, a large percentage of minority participants (69%), and introduction to an innovative online resource and technology hub. Although there were a couple of factors influencing the Post-CC, our data support the hypothesis that there are significant gains as a result of the utilization of the virtual collaboratory space.

Although six months appears to be a rather short timeframe, the analysis was standardized for this period to react to the nuances with the individual coaching groups. Throughout the course of the coaching group sessions, many participants were submitting grants that were not previously indicated in their application. For example, if an ESI came into the program intending to submit an R01, they sometimes chose to submit a K grant proposal instead. This change was the result of an overestimation of their readiness to submit through a specific mechanism. After discussions in their coaching group, their coach sometimes advised them to consider a funding mechanism that was more consistent with their career stage and goals, to increase their competitiveness for funding. Since we saw that ESIs’ mechanisms were slightly different based on discussions with and expert advice from their primary coach, it was important to capture these submissions and the time it took to submit their initial proposal. This submission outcomes occurred within the 6 months allotted for the intervention and therefore produced 6-month submission data. Since this longitudinal study occurs on an assessment schedule, the 12-month data for the post collaboratory cohort will be collected and longer-term data is will be available in future dissemination.

The SETH provides many unique opportunities (both in-person and online) for health disparities ESI to develop their research skills and networks, including career-development seminars, grant writing training, health disparities research seminars on team science, community engagement, translational science, and networking. Scholars noted the benefits of this program in the EQ-Collaboratory: “[This] NMRN program gives a chance to those stemming from a lack of mentoring opportunities and from departments without an established research mentoring infrastructure. The knowledge and support provided already at the initial meeting and from our small-group meetings in the EQ-Collaboratory gives confidence, motivates, and prepares us to submit competitive grants to NIH.” ESIs have also had a positive experience with the overall grant writing program noting, “The program has really been very helpful to new investigators like me to present all components of the proposal with clarity, and [it is] innovative enough for funding opportunities. I have so far written four grants in which one is under review and the rest yet to be submitted. Your coaches are very excellent.” These responses show the overall benefits ESIs received from engagement with not only the program but also the EQ-Collaboratory community.

Although the program had many benefits as a byproduct of participation, this study revealed that merely introducing a trainee to the content and knowledge of a grant writing coaching group was insufficient to increase the likelihood of proposal submission by these scholars. Consequently, introduction to a virtual community and an expanded network may have helped to improve submission habits and decrease the time to submission of the grant proposal.

In our future research plans, we will follow these participants for a longer period to assess the impact on their grant writing skills, the likelihood of future grant submissions, grants awarded, and career progression. Since 18-month follow-up data were available only for trainees in the pre-collaboratory cohort 1, future studies should randomize participants, collect and explore 18-month follow-up data for both cohorts to determine submission and award rates for pre- and post-collaboratory groups. Additionally, based on the preliminary data presented here, future studies should address the additional research questions regarding submission timeframe and likelihood of award. Analyzing and comparing these longitudinal data will provide a transparent perspective and offer insight into the submission habits over a longer timeframe.

## 5. Conclusions

The collaboratory is a strategy to improve productivity and impact career outcomes as measured by the time to prepare for grant proposal submissions and submission rates. The significant differences in the outcomes lead us to believe that the collaboratory could contribute to submission tendencies because of the social and developmental support it offers. Additionally, decreased time to submission also allows underrepresented ESIs to focus more of their attention on their research programs and on preparing subsequent applications for funding. Therefore, investigators may prepare more grants in the hopes of equally successful funding outcomes with respect to their well-represented peers. This online platform can be a cost-effective solution that could potentially overcome resource and geography barriers for URG faculty career development.

## Figures and Tables

**Table 1:** Comparison of Likelihood to Submit in 6 months: Pre- and Post-Collaboratory Cohorts.

Variable	Odds Ratio (OR)	95% CI	*p*-Value
Cohort	Unadjusted OR		
Ref: Pre-Collaboratory	1.00		
Post-Collaboratory	2.89	1.305–6.42	0.009
Cohort	Adjusted OR		
Ref: Pre-Collaboratory	1.00		
Post-Collaboratory	4.828	1.745–13.357	0.002
